# The development of an extra-anatomic tissue-engineered artery with collateral arteries for therapeutic angiogenesis in ischemic hind limb

**DOI:** 10.1038/s41598-018-22799-4

**Published:** 2018-03-15

**Authors:** Xu Zhou, Yinlong Zhang, Hongfei Wang, Bin Zhao, Jinling Wang, Guoliang Yan, Shuangyue Xu, Yuanyuan Zhou, Hongyi Liu, Yifei Zheng, Wei Quan, Jianyin Zhou, Yun Liu, Maochuan Zhen, Xuan Zhu, Yilin Zhao

**Affiliations:** 1 Oncology and Vascular Interventional Radiology, Zhongshan Hospital, Xiamen University, Xiamen, Fujian Province P. R. China; 2 Medical College, Xiamen University, Xiamen, Fujian Province P. R. China; 3 Vascular surgery, Changhai Hospital, Second Military Medical University, Shanghai, P. R. China; 4 Emergency, Zhongshan Hospital, Xiamen University, Xiamen, Fujian Province P. R. China; 5 Basic Medical Department of Medical College, Xiamen University, Xiamen, Fujian Province P. R. China; 6 Organ Transplantation Institute, Xiamen University, Fujian Key Laboratory of Organ and Tissue Regeneration, Xiamen, Fujian Province P. R. China; 7 Hepatology Surgery, Zhongshan Hospital, Xiamen University, Fujian Provincial Key Laboratory of Chronic Liver Disease and Hepatocellular Carcinoma, Xiamen, Fujian Province P. R. China; 8 College of Pharmacy, Xiamen University, Xiamen, Fujian Province P. R. China

## Abstract

To develop tissue-engineered arteries (TEAs) with collateral arteries(CAs) in ischemic hind limb goat models(IHLMs). The IHLMs created by removing femoral arteries were divided into non-treated control group(NG); non-catheter group (NCG) in which TEA was anastomosed to external iliac artery(EIA), and surrounded with collagen sponge containing autologous MSCs and VEGF-gelatin microspheres, the distal end of TEA was ligated; catheter group(CG) which received the same procedure as NCG, also received heparin infusion through catheter in EIA. TEA patency was assessed weekly by Ultrasound. The TEA and CAs were assessed by angiography, gross examination, histology and electron microscopy. In CG, TEAs remained patent for 1 month, but became partly occluded 1 week after catheter withdrawn. In NCG, TEAs were occluded 1 week after implantation. Angiography demonstrated that communication between CAs arising from the TEAs and the native vessels was established in both groups. NCG had fewer CAs than CG (P < 0.01). At 40 days, TEAs in CG demonstrated of endothelium formation, smooth muscle cells infiltration and collagen regeneration. The CG had more capillaries and mature vessels in adventia of TEAs than NCG (P < 0.01). CG group also had more vessels around TEAs than NCG (P < 0.01) or NG (P < 0.001).

## Introduction

A large number of chronic peripheral artery disease (PAD) patients are not candidates for the conventional revascularization techniques of angioplasty, stenting, and peripheral artery bypass grafting^1–4^, but advances continue to be made in the prevention and treatment of PAD^5–7^. Alternative noninvasive methods now available include localized delivery of therapeutic growth factors^8,9^and autologous stem cell transplantation^10^.

The administration of VEGF and bFGF have demonstrated beneficial results in reversing ischemia and improving regional blood perfusion in extremities in both animal and clinical trials^11^. However, growth factors such as VEGF or bFGF have poor stability *in vivo* and consequently, short-lasting biological activity^12^. Sustained-release systems, with gelatin as the drug carrier^13–15^, have been developed that ensure gradual, localized delivery of growth factors over the span of a few weeks.

However, questions remain regarding the efficacy of treatment with such systems, especially in patients who have advanced PAD with many occluded arteries. For example, after revascularization has been induced with the administration of angiogenic factors, what will be the source of blood supply to the new collateral vessels?

One approach might be to combine the Vineberg procedure, which has been virtually neglected for decades, with therapeutic angiogenesis^16^. In animal experiments, Arthur Vineberg dissected the internal thoracic artery from the chest wall, ligated the distal end, and pulled it into a tunnel created in the superficial myocardium^17^. He found that the artery sprouted new collateral vessels that connected with the native myocardial blood vessels^18^. The Vineberg operation has been used as a last resort in patients with diffuse atheromatous lesions who are unsuited for direct revascularization with methods such as angioplasty, stenting, and bypass surgery^19^.

However, the Vineberg procedure is not suitable for PAD patients, as autologous arteries are unavailable for use in this location. Completely synthetic grafts, such as Dacron® and ePTFE vascular grafts, are also ruled out as they cannot develop collateral branches to establish communication with the native vasculature in the ischemic extremity. Tissue-engineered (TE) blood vessels, with decellularized vascular grafts used as a scaffold, could be a possible solution; this acellular tissue retains its natural mechanical properties and facilitates neovascularization and recellularization after transplantation^20^.

Therefore, in this study, we applied the Vineberg procedure in an ischemic hind limb animal model, using a TE artery surrounded by collagen complex containing autologous MSCs and gelatin microspheres formulated for sustained VEGF release^21–23^. We studied whether this extra-anatomic artery could develop collateral artery branches to communicate with local vessels and recover the blood supply to the ischemic hind limb.

## Methods

### Preparation and characterization of nanosized decellularized artery scaffolds (NDAs)

Fresh goat carotid arteries, with lumen diameters of 4.0 ± 0.2 mm, were harvested from the local slaughterhouse. The procedure for preparation and examination of NDAs, the DNA quantification and mechanical testing were based on our previous published work^24,25^. The 5-0 polypropylene sutures (Ethilon, USA) were placed in each side of the scaffold at 1 mm from the edge. A constant elongation (5 mm/min) was applied along the longitudinal axis of the scaffold until the sutures were pulled through the vessel edge, and the maximum suture strength was recorded by Instron mechanical tester (Norwood, MA). Fifty-millimeter scaffold samples were attached via tapered adapters directly into the pressurized tube and tensioned and submerged in distilled water. The system was progressively pressurized until vessel failure when air bubbles from the water can be observed. The burst pressure was recorded by a manometer with data acquisition system (Testo, Germany). The porosity of the scaffold was measured using the following formula: Porosity (%) = (bulk volume − true volume)/bulk volume × 100%. The true volume of scaffold were analyzed using True Density Analyzer with helium gas (Beijing Builder Electronic Technology, China). Bulk volume of the scaffold was calculated by measuring volume.

### Preparation and characterization of gelatin microspheres (GMs)

Gelatin (2 g; Sigma-Aldrich, St Louis, MO, USA) was dissolved in15 ml phosphate-buffered saline (PBS; pH 7.4). The preparation and examination of GMs as described by our previous our previous published work^26,27^.

### Preparation and characterization of collagen scaffolds (CSs)

Type I collagen (100 mg; Sigma-Aldrich) was mixed in 40 ml 0.1% acetic acid solution at 2000 rpm for 30 min to prepare collagen protein solution. The preparation and examination of CSs as described by our previous our previous published work^24,28^.

### Rehydration analysis

Dried samples of NDAs, GMs, and CSs were immersed in PBS. After 24 h, the samples were removed from the solution and the surface was blotted dry with a piece of tissue; the samples were then weighed.

### Cytotoxicity of NDAs, GMs, and CSs

A 96-well microplate containing 200 μl culture medium per well was taken and seeded with mesenchymal stem cells (MSCs) at a density of 1.0 × 104 cells per well. This was kept at 37 °C in a 5% CO_2_ atmosphere for 24 h, after which the culture medium was replaced with fresh medium containing 200 μg/ml of NDAs, GMs, or CSs pieces; normal medium was used as a control. After 1, 3, 7, and 9 days, the medium was removed and the cells were detached by 0.05% trypsin with ethylene diamine tetraacetic acid (EDTA), and3-(4,5-dimethyl-2-thiazolyl)-2, 5-diphenyl-2-H-tetrazolium bromide (MTT)assay was performed.

### Incorporation of VEGF in GMs (GMs-VEGF)

Recombinant human VEGF (rhVEGF; Applied Biosystems, California, USA) solution (20 μg/mL) was dropped onto the GMs. The GMs-VEGF was kept at room temperature for 4 h and then lyophilized for 24 h.

### Isolation of MSCs

MSCs were isolated from goat bone marrow (Xiamen University Animal Center). The procedure for preparation of MSCs was based on our previous published work^25^. Briefly, bone marrow aspirated from the anterior iliac crest was diluted with heparinized phosphate-buffered saline (PBS). Ficoll density gradient centrifugation was used to separate MSCs from other cells. The residual cells were washed three times with PBS and incubated at 37 °C, 90% humidity, and 5% CO_2_. Second-passage cells at subconfluence were used for all experiments. All the cells used for experiments were labeled with PKH67(Sigma-Aldrich).

### Characterization of MSCs

The immunophenotype of MSCs was determined by flow cytometry according to previous described methods^29,30^. Second-passage MSCs were harvested, non specific binding was blocked and the cells were stained with different antibodies against the human antigens: CD29-FITC, CD90- FITC and CD45-FITC(eBioscience, California, USA). Nonspecific isotype matched antibodies were used as controls. The fluorescence intensity of the cells was evaluated by flow cytometer and the data were analyzed with the CytExpert software (Beckman Coulter, California, USA). Results are presented as percent positively stained cells.

### Incorporation of MSCs and GMs-VEGF into CSs (CSs complex)

MSCs and GMs-VEGF were seeded into collagen sponges by the agitated seeding method. Collagen complex (1 × 1 × 0.3 cm^3^) was placed into a dish, and 1 × 10^6^ MSCs in 1 ml suspension and 50 mg GMs-VEGF were evenly injected into the CSs complex by blunt-end needle syringes. The collagen complex was placed in a 50 ml tube and agitated at 300 rpm on an orbital shaker for 6 h at 37 °C. The complex was then incubated with 2 ml DMEM supplemented with 10% FBS and 100 u/ml penicillin–streptomycin in a dish for 2 h at 37 °C. The collagen complex was routinely examined under light and fluorescence microscopes; H&E staining and SEM were used for further evaluation of the collagen complex. The MSCs proliferation within the collagen complex was assessed at designated intervals using MTT assay.

### *In vitro* release of VEGF

GMs-VEGF and CSs-GMs-VEGF specimens were placed in 5 ml centrifuge tubes containing 2 ml PBS (pH 7.4). The tubes were maintained at 37 °C with gentle shaking for up to 14 days. At the designated time intervals, the tubes were taken out and centrifuged at 12 × 10^3^ rpm for 15 min, and the supernatant was transferred into a test tube and stored at −20 °C till VEGF analysis was performed. Equal amount of fresh PBS was added to the original tubes till the next measurement. The amount of released VEGF was analyzed using a human VEGF ELISA kit(Abcam, Cambridge, UK) according to the manufacturer’s instructions.

### Tissue-engineered artery preparation

MSCs suspensions (1 × 10^6^ cells/ml) were stained with PKH67 (Sigma-Aldrich) and seeded into the 3 cm long NDAs. The constructions were cultured for 9 days, and observed under fluorescence microscopy. MSCs proliferation was analyzed by MTT assays at regular intervals.

### Vineberg procedure

Goat experiment was approved by Laboratory Animal Management Ethics Committee of Xiamen University. Animal care and experimental procedures were carried out strictly in compliance with the Guide for the Care and Use of Laboratory Animals published by the National Institutes of Health(NIH Publications No. 8023, revised 1978).

The adult goats (mean weight, 20 ± 2 kg) were anesthetized with intramuscular ketamine (30 mg/kg) and intravenous pentobarbital (30 mg/kg) and ventilated with a mixture of O_2_, N_2_, and isoflurane during the operation. The acute ischemic hind limb goat model was created by ligating the external iliac and popliteal arteries, and removing the femoral artery between the ligatures. The goat models were divided into a catheter group (CG; n = 3), a non-catheter group (NCG; n = 3), and a control group (NG; n = 3). The control group did not receive any treatment. In the non-catheter group, the prepared TE artery was anastomosed to the external iliac artery using 6-0 polypropylene sutures (Ethicon, USA). The distal end was ligated and surrounded with CSs containing autologous MSCs and sustained-release GMs-VEGF formulations. In the catheter group, the same procedure was performed but, in addition, a catheter was placed in the external iliac artery for continuous heparin infusion (at the rate of 5 u/kg/h) to maintain anticoagulation within the TE artery. No anticoagulants were used in the other groups. The implanted TE vascular conduits were approximately 30 mm in length and had an average inner diameter of 4 mm.

### Monitoring, evaluation, and harvest of the graft

To determine graft patency after implantation, the animals were examined by Doppler ultrasound every week. Angiography was performed 1 month after surgery, and the catheter (in the catheter group) was removed after angiography. All animals were sacrificed at the end of 40 days for gross examination and histological analysis of the graft. The collateral vessels arising from the TE artery wall were counted on the gross specimen and in the angiography images.

### Histological and immunohistochemical examination

Before implantation, the NDAs, CSs complex, and TE artery were all histologically examined after H&E, Masson’s trichrome, and Van Gieson’s staining. Segments of the implanted vascular graft and surrounding tissue were harvested 40 days after implantation, fixed in 10% buffered formaldehyde solution, dehydrated with a graded ethanol series, embedded in paraffin, cut into 5-μm-thick sections, and stained with H&E. Masson’s trichrome and van Gieson’s stains were used to examine collagen and elastin deposition in the harvested vascular conduits. Immunostaining for von Willebrand Factor (vWF) and α-Smooth Muscle-Actin (α-SMA) was also performed; brown granular deposits indicated the presence of vWF or α-SMA. The vessels per unit area were counted in five randomly chosen fields per slide of each portion by three blinded pathologists. The average number of vessels in one portion was used for assessment of vascular number. Vessels with diameter >50 μm were considered mature.

We harvested the samples at proximal, middle and distal part of TE arteries and got the samples at 0, 4 and 8 o ‘clock points at each part. The sections of specimens were stained with immunostaining for vWF, the coverage of endothelium in TE arteries was calculated according to the formula (the length of positive vWF staining/total detected length %) in five randomly chosen fields per slide by three blinded pathologists.

### *In vivo* differentiation of MSCs

The samples were fixed and cut into 5-µm-thick sections. The slides were examined for fluorescence to trace the implanted MSCs, and then incubated with vWF antibody and FITC-conjugated secondary antibody (Boster, Wuhan, China) to detect endothelial cells. The signals were visualized using a confocal laser scanning microscope (Leica, Wetzlar, DE).

### Electron microscopy

For SEM, specimens were fixed in 1% buffered glutaraldehyde and 0.1% buffered formaldehyde for 1 and 24 h, respectively, dehydrated with a graded ethanol series, and dried. The dried samples were mounted on an aluminum stub and coated with gold using a sputter coater before SEM (TESCAN, Czech Republic).

### Statistical analysis

The suture retention test and burst pressure test were repeated at least three times. Data were expressed as means ± SD. The Student’s t test was used for comparison between groups. Statistical significance was at P < 0.05.

## Results

### Nanosized fiber decellularized scaffolds

**Figure 1:**
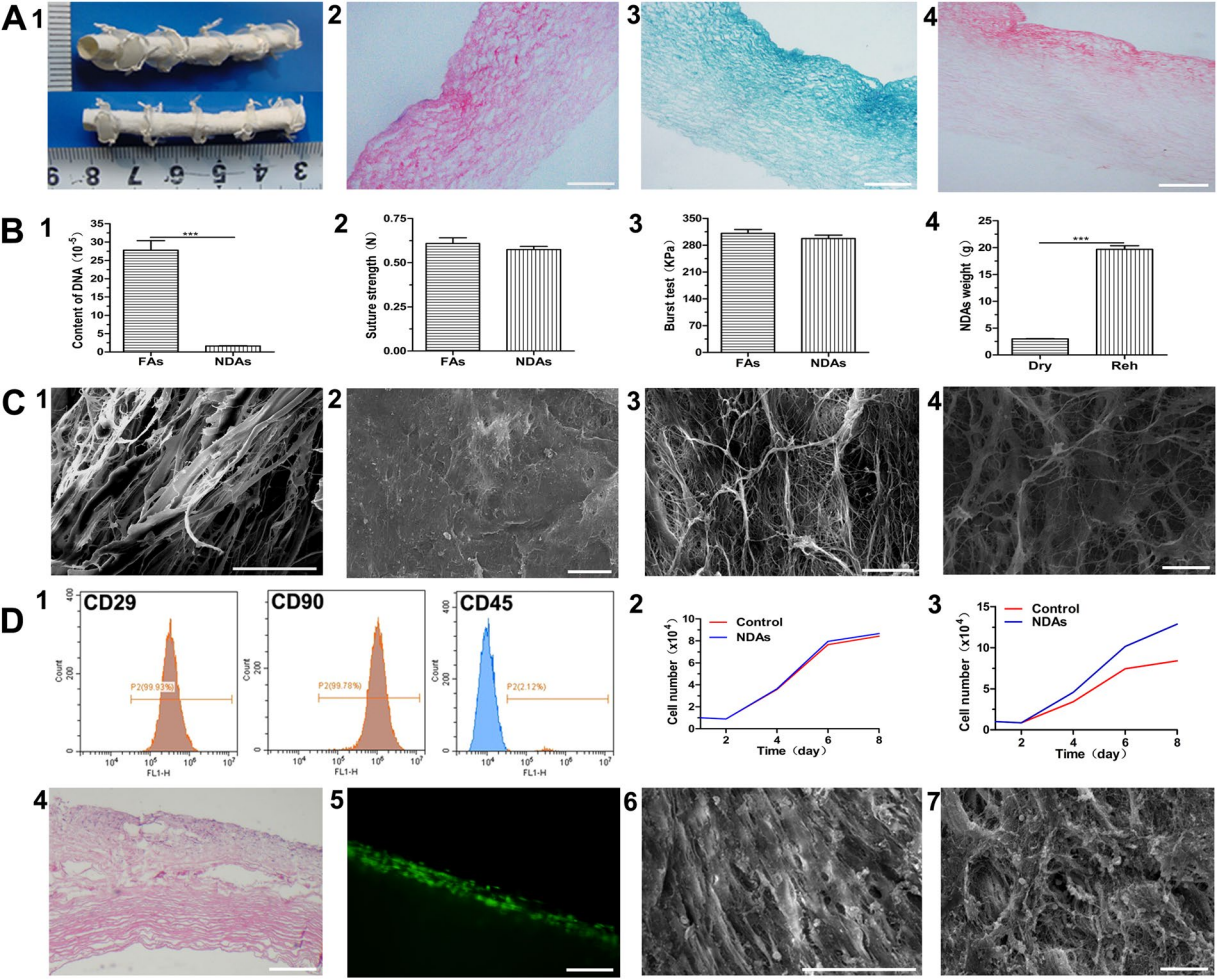
Nanosized decellularized arteries (NDAs). (**A**) Biological structures, (1) Gross view, (2) H&E (bar: 200 μm), (3) Masson’s trichrome staining (bar: 200 μm), (4) Van Gieson’s staining (bar: 200 μm). (**B**) Test, (1) DNA content (*P* < 0.001), (2) Suture strength (*P* > 0.05) Fresh arteries (FAs), (3) Burst pressure (*P* > 0.05), (4) Rehydration (*P* < 0.001). (**C**) Microstructure, (1) Cross-section of NDAs (SEM, bar: 50 μm), (2) Intima (SEM, bar: 10 μm), (3) Subintima and media (SEM, bar: 10 μm), (4) Adventitia (SEM, bar: 10 μm). (**D**) Cell seeding, (1) Immunophenotype of MSCs by flow cytometry, (2) Cytotoxicity (*P* > 0.05), (3) Cell proliferation on NDAs, (4) MSCs (green) seeded NDAs (H&E, bar: 200 μm), (5) MSCs seeded NDAs (bar: 100 μm), (6) Intima of MSCs seeded NDAs (SEM, bar: 50 μm), (7) Adventitia of MSCs seeded NDAs (SEM, bar: 10 μm).

The NDAs were white, with lumen diameter of 4.0 ± 0.5 mm and wall thickness of about 200 ± 25 μm (Fig. 1A_1_). All NDAs retained their original shape in PBS. H&E staining showed that all cellular components had been completely removed from the fresh arteries and that the remaining acelluar scaffold was a multilayer structure(Fig. 1A_2_). Masson’s trichrome staining (Fig. 1A_3_) and Van Gieson staining (Fig. 1A_4_) demonstrated the existence of collagen and elastin, respectively, throughout the scaffold. Masson’s trichrome staining also confirmed that smooth muscle and cytoplasm had been totally removed in the NDAs. DNA content was 1.62 ± 0.11 × 10^−5^ in NDAs vs. 27.78 ± 2.62 × 10^−5^ in fresh artery (Fig. 1B_1_). Mechanical testing showed no significant difference in suture retention between fresh artery and NDAs (0.61 ± 0.03N vs. 0.57 ± 0.02N, respectively; P > 0.05; Fig. 1B_2_). Burst pressure was also comparable between NDAs and fresh artery (297.0 ± 9.3 KPa vs. 310.9 ± 9.9 KPa, respectively; P > 0.05; Fig. 1B_3_). After rehydration, the NDAs showed a 6-fold increase in weight (P < 0.001; Fig. 1B_4_). SEM confirmed that no cells were left within the NDAs, and that the architecture mainly consisted of nanoscale- to microscale-sized collagen fibers. The cross-section of the NDAs revealed a porous structure with a uniform pore size. In the cross-sections the average distance between collagen fibers was about 20 ± 5 μm (Fig. 1C_1−4_).

MSCs exhibited similar fibroblastic morphology in normal culture. Flow cytometry analysis demonstrated that most MSCs were positive for CD29(99.93%) and CD90(99.78%) and negative for CD45(97.88%) (Fig. 1D_1_). The proliferation rate of MSCs in medium containing NDAs was not significantly different from the rate in control medium (Fig. 1D_2_). The porosity and variations in the size of the collagen fibers provided a three-dimensional space which was more conducive for MSCs proliferation than a plain dish (P < 0.05; Fig. 1D_3_). H&E staining (Fig. 1D_4_), fluorescent detection (Fig. 1D_5_), and SEM (Fig. 1D_6_) demonstrated MSCs adhesion and proliferation within the NDAs. Moreover, MSCs were also shown to secrete nanosized fibers of extracellular matrix (ECM) on the NDAs (Fig. 1D_7_).

### Gelatin microspheres, collagen scaffold, and the CSs complex

**Figure 2:**
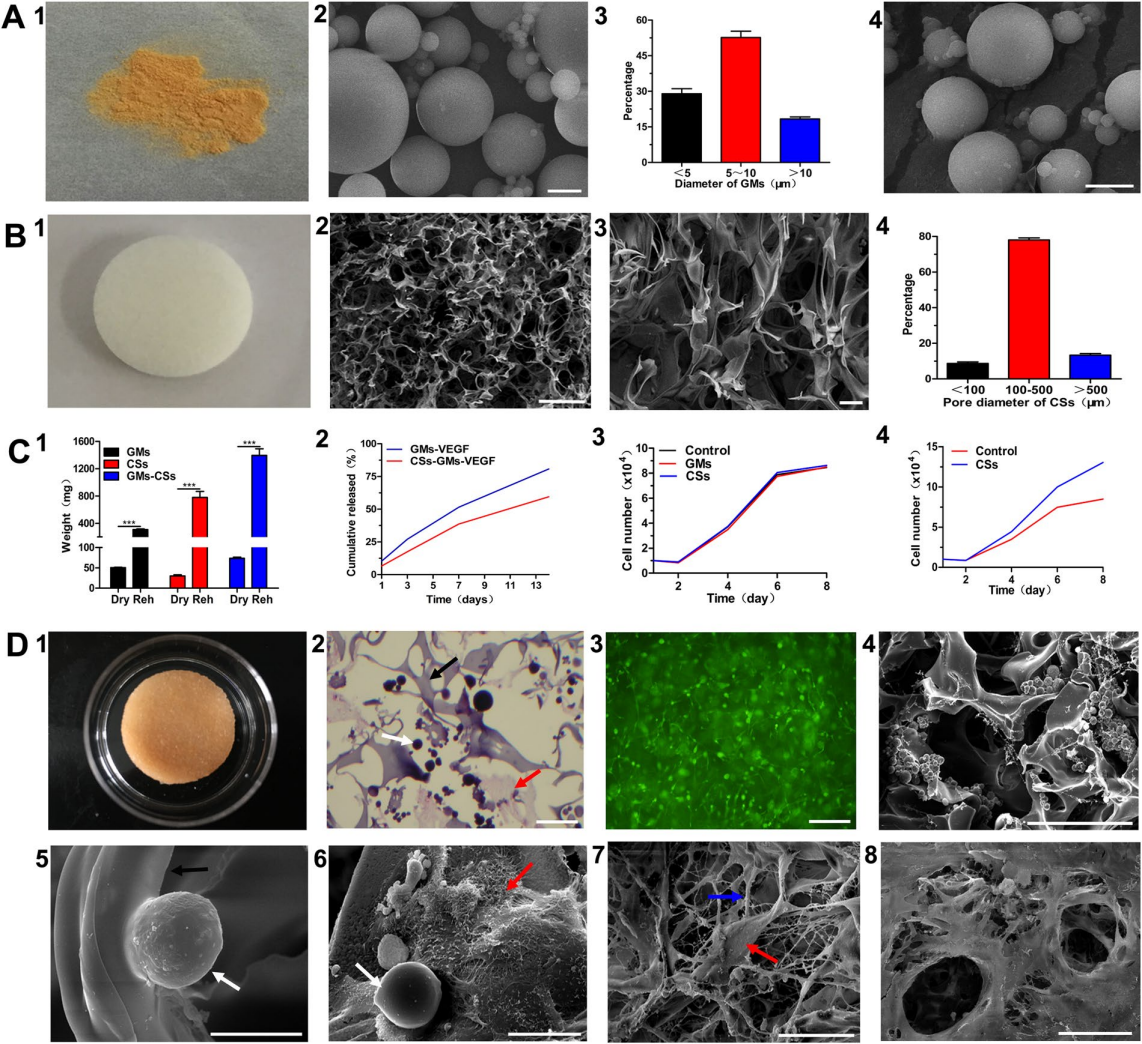
sustained-releasing system. (**A**) GMs, (1) Gross appearance, (2) SEM (bar: 5 μm), (3) Size distribution, (4) GMs-VEGF (SEM, bar: 5 μm). (**B**) CSs, (1) Gross appearance, (2) SEM (bar: 500 μm), (3) SEM (bar: 10 μm), (4) Distribution of pore size. (**C**) Character, (1) Rehydration of GMs, CSs and GMs-CSs (P < 0.001), (2) VEGF Releasing of from GMs-VEGF and CSs-GMs-VEGF (*P* < 0.05), (3) Cytotoxicity of GMs and CSs (*P* > 0.05), (4) Cell proliferation on CSs. (**D**) CSs complex (GMs-MSCs-CSs), (1) Gross appearance, (2) H&E (bar: 50 μm), (3) MSCs (green) proliferated on the NDAs (bar: 50 μm), (4) SEM (bar: 200 μm), (5) GMs in the CSs complex (SEM, bar: 20 μm), (6) MSCs and GMs in CSs complex (SEM, bar: 20 μm), (7) MSCs and ECM secreted by MSCs in CSs complex (SEM, bar: 50 μm), (8) ECM of MSCs (SEM, bar: 100 μm), CSs (Black arrow), GMs (White arrow), MSCs (Red arrow), ECM (Blue arrow).

On gross examoniation, GMs appeared as a yellow, well-dispersed powder (Fig. 2A_1_). On SEM, the GMs were spherical, with an average diameter of 10 ± 5 μm (Fig. 2A_2_); the diameter was <5 μm in 29.0% of GMs, 5–10 μm in 52.7%, and >10 μm in 18.3% (Fig. 2A_3_). After rehydration, the GMs showed a 6-fold increase in weight (P < 0.001). For the incorporation of VEGF in GMs, entrapment efficiency of 89.0 ± 2.7%. The spherical shape of the GMs showed no change after loading VEGF (Fig. 2A_4_). The collagen scaffold was white, spongy, multiporous, and soft (Fig. 2B_1–3_). The pore size was mostly (78.0%) between 100 and 500 μm (Fig. 2B_4_). After rehydration the CSs showed a 25-fold increase in weight (P < 0.001; Fig. 2C_1_).We analyzed the VEGF release profile of GMs-VEGF and CSs-GMs-VEGF over a period of 14 days (Fig. 2C_2_). The VEGF release was significantly slower in CSs-GMs-VEGF than in GMs-VEGF (P < 0.01). Release from GMs-VEGF occurred in a sudden burst, with 27.10% of VEGF being released during the first 3 days. At the end of 14 days, 80.80% had been released. In contrast, VEGF release from CSs-GMs-VEGF presented a sustained profile, with only 17.57% of VEGF being released in the first 3 days; at the end of 14 days, 59.57% of the impregnated VEGF had been released. MSCs proliferation assays were used to evaluate the cytotoxicity of GMs and CSs. The proliferation rate of MSCs cultured with GMs or CSs was not significantly different from that of MSCs cultured in control medium (Fig. 2C_3_). MSCs proliferation was significantly faster on the CSs than in a plain dish (P < 0.01; Fig. 2C_4_), indicating that the three-dimensional architecture of CSs is more suitable for cellular adhesion and proliferation.

After multipoint injection seeding and agitation, GMs-VEGF and MSCs were evenly distributed within CSs (Fig. 2D_1_). The density of GMs-VEGF was about 34.0 ± 1.4 mg/cm^3^. H&E staining (Fig. 2D_2_) and fluorescence microscopy (Fig. 2D_3_) showed that GMs and MSCs were uniformly distributed within the CSs. SEM showed that the GMs and MSCs were evenly distributed in the pores of the CSs (Fig. 2D_4_), Both GMs (Fig. 2D_5_) and MSCs (Fig. 2D_6_) were found attached to the fibers of the CSs. SEM also demonstrated that MSCs secreted nanosized extracellular matrix fibers, which connected with the structure of CSs (Fig. 2D_7–8_).

### TE artery patency and collateral artery development

**Figure 3:**
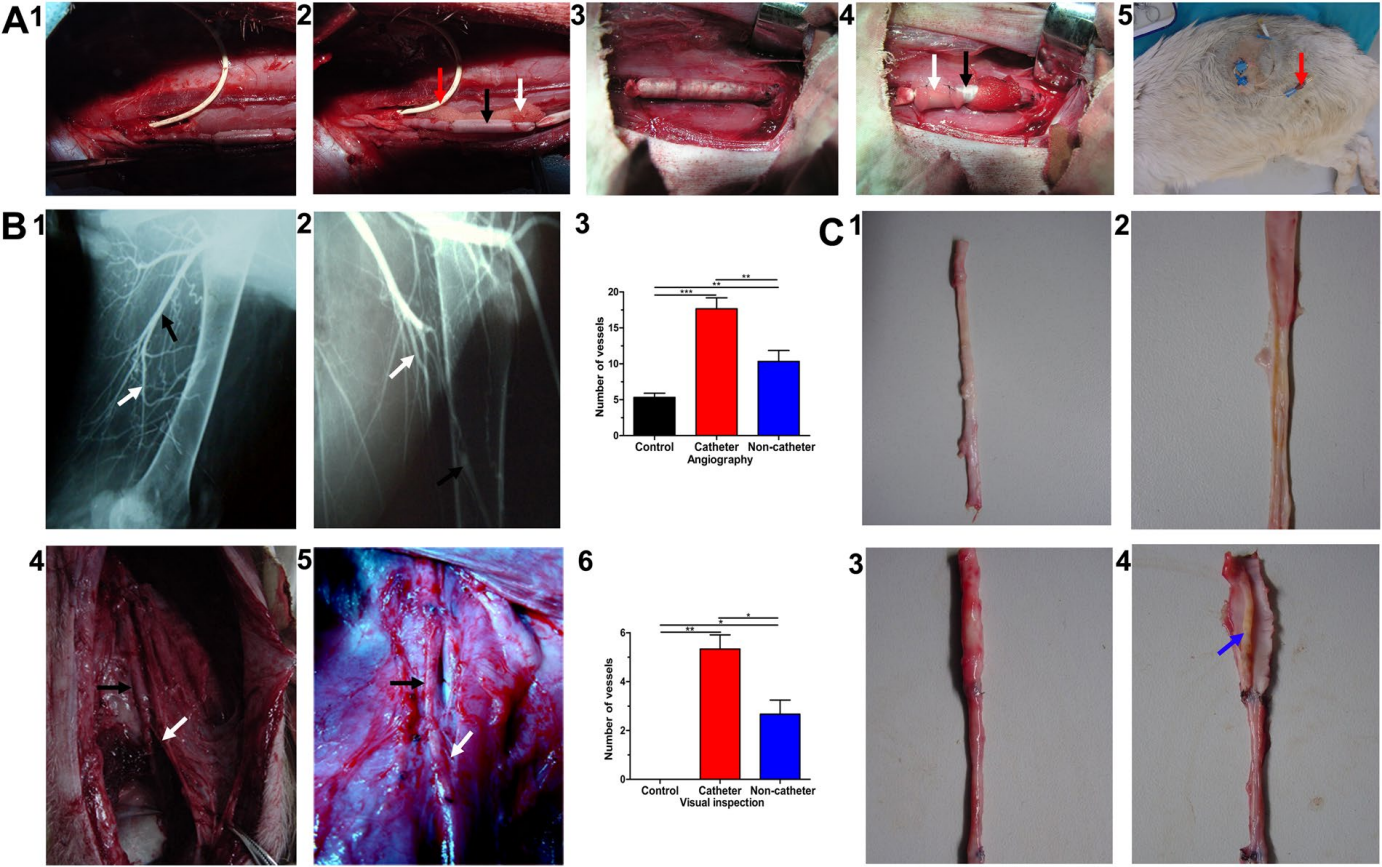
Implantation and following examination. (**A**) Implantation, (1) TE artery in CG, (2) TE artery was surrounded by CSs complex in CG, (3) TE artery in NCG, (4) TE artery was surrounded by CSs complex in NCG, (5) The catheter secured on the skin for heparin infusion. (**B**) Angiography and anatomy, (1) Angiography of CG, (2) Angiography of NCG, (3) Collateral arteries (CAs) in NG, NCG and CG by angiography, (4) CAs arising from TE artery in CG, (5) CAs arising from TE artery in NCG, (6) Quantification of CAs. (**C**) Gross examination, (1) TE artery in CG, (2) Intima of TE artery in CG, (3) TE artery in NCG, (4) Thrombosis of TE artery in NCG, TE artery (Black arrow), CA (White arrow), catheter (Red arrow) and thrombosis (Blue arrow).

Angiography was performed to assess the patency of the implanted TE arteries and collaterals formation. While the TE arteries in the non-catheter group presented thrombosis and occlusion by the seventh day after transplantation, the arteries in the catheter group were still patent at 1 month after surgery. Many collateral arteries were visible around the TE arteries (Fig. 3B_1_). Collateral arteries were also visible in the non-catheter group although the grafts were completely occluded (Fig. 3B_2_). Angiography revealed 5 ± 1 collateral blood vessels in the control group, 10 ± 2 in the non-catheter group, and 18 ± 2 in the catheter group (Fig. 3B_3_); the difference between the groups was statistically significant (P < 0.01). With the assumption that the patent TE arteries in the catheter group had achieved endothelial integrity, we removed the catheters. However, 1 week after catheter removal, sonography showed that the TE arteries had become occluded.

The TE arteries were harvested at 40 days after transplantation. In both the catheter and the non-catheter groups, the CSs-GMS-VEGF-MSCs complex was found to have completely degraded. The TE arteries were closely integrated with the surrounding tissue. Dissecting or separating the TE arteries from the surrounding tissue was difficult and caused obvious bleeding. In the catheter group, many collateral arteries had formed from the wall of the TE artery. The collateral vessels were obvious on gross view, and angiography had earlier demonstrated contrast perfusion from the TE arteries and their collateral vessels into the native distal vessels (Fig. 3B_4_); unfortunately the TE arteries had become occluded and thrombosed after the heparin infusion catheter was pulled out. The non-catheter group also showed collateral vessels formation around the TE arteries (Fig. 3B_5_). On gross observation there were significantly more collateral branches arising from the TE arteries in the catheter group (5 ± 1 vs. 3 ± 1 in the non-catheter group; P < 0.05); no new blood vessels were visible in the control group (Fig. 3B_6_). The TE arteries in the catheter group had smooth luminal surfaces (Fig. 3C_1–2_), but in the non-catheter group the lumina were completely occluded (Fig. 3C_3–4_).

### TE artery regeneration and its adventitial angiogenesis

**Figure 4:**
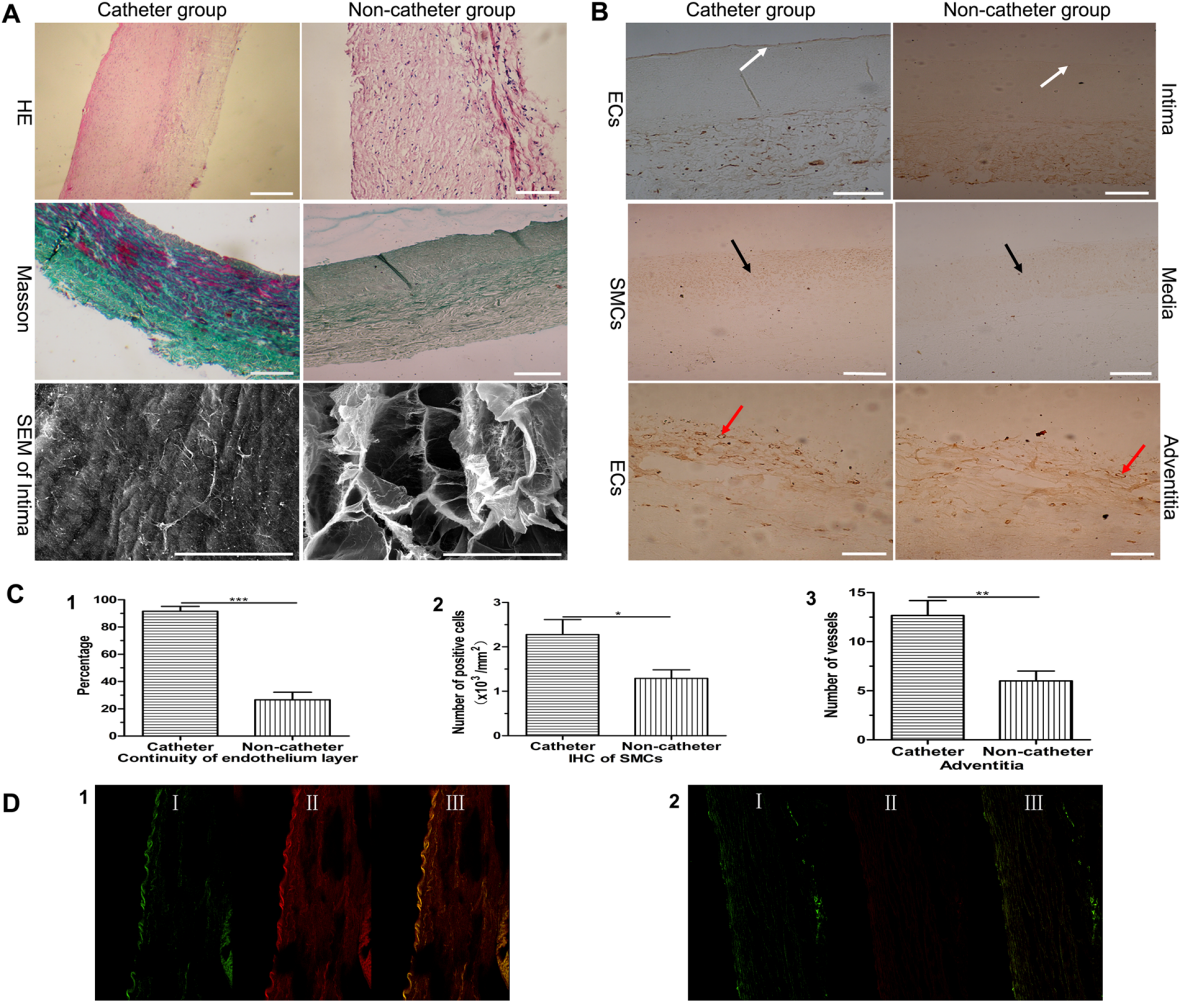
Regeneration of TE artery in CG and NCG. (**A**) H&E (bar: 200 μm), Masson’s trichrome (bar: 200 μm) and Intima of TE artery (SEM, bar: 50 μm in CG, bar: 100 μm in NCG). (**B**) Endothelial layer, smooth muscle cell layer and vascularization within the adventitia of TE arteries (IHC, bar: 100 μm). (**C**) Quantification, (1) Continuity of endothelial layer, (2) Quantification of SMCs of TE artery, (3) Quantification of vessels formation in the adventitia of TE artery. (**D**) Colocalization of MSCs and ECs (I green indicated MSCs, II red showed ECs, III merge), (1) Intima of TE artery in CG, (2) Intima of TE artery in NCG, Endothelium (White arrow), SMC (Black arrow), blood vessels of adventitia (Red arrow).

In the catheter group, H&E stained sections of the TE arteries showed the presence of all three layers of the arterial wall (endothelium, media, and adventitia). Masson’s trichrome staining confirmed the presence of regular collagen and smooth muscle throughout the vascular graft, and SEM confirmed that the endothelium at 40 days post-implantation was similar to the native endothelium. In the non-catheter group, H&E staining of TE arteries showed that the three-layer structure of the arterial wall was incomplete. Masson’s trichrome staining showed irregular laying down of collagen, and SEM demonstrated an incomplete and rough endothelium (Fig. 4A). On immunohistochemical analysis the luminal surface of the TE arteries in the catheter group was positive for vWF, indicating endothelial regeneration, and the medial layer was positive for α-SMA, indicating smooth muscle regeneration. Relatively complete smooth muscle cell layer has been formed in the TE artery of the catheter group. In the non-catheter group, it shows the poor endothelial regeneration on the luminal surface and the scattered and disorderly arrangement of smooth muscle cells in TE artery (Fig. 4B).

On examination of the luminal surface of the TE arteries, 91.7% was covered by endothelial cells in the catheter group vs. 26.7% in the non-catheter group (Fig. 4C_1_). There were about 2.275 ± 0.338 × 10^3^ cells/mm^2^ in the catheter group vs. 1.292 ± 0.191 × 10^3^ cells/mm^2^ in non-catheter group (Fig. 4C_2_). Blood vessel formation in the adventitia of TE arteries was more in the catheter group (13 ± 2 vs. 6 ± 1 in the non-catheter group (Fig. 4C_3_). The results of immunofluorescent staining also supported these findings. The endothelial cell lining on the intimal surface of the TE arteries in the catheter group (Fig. 4D_1_) was almost continuous, whereas it was discontinuous in the non-catheter group (Fig. 4D_2_). The data also indicated that some implanted green fluorescent MSCs on the intima of TE arteries of catheter and non-catheter group were co-localized with red fluorescent endothelial cells on the intima. The results proved that MSCs differentiated into endothelial cells.

### Angiogenesis, vasculogenesis, and arteriogenesis in the tissue around the TE artery

**Figure 5:**
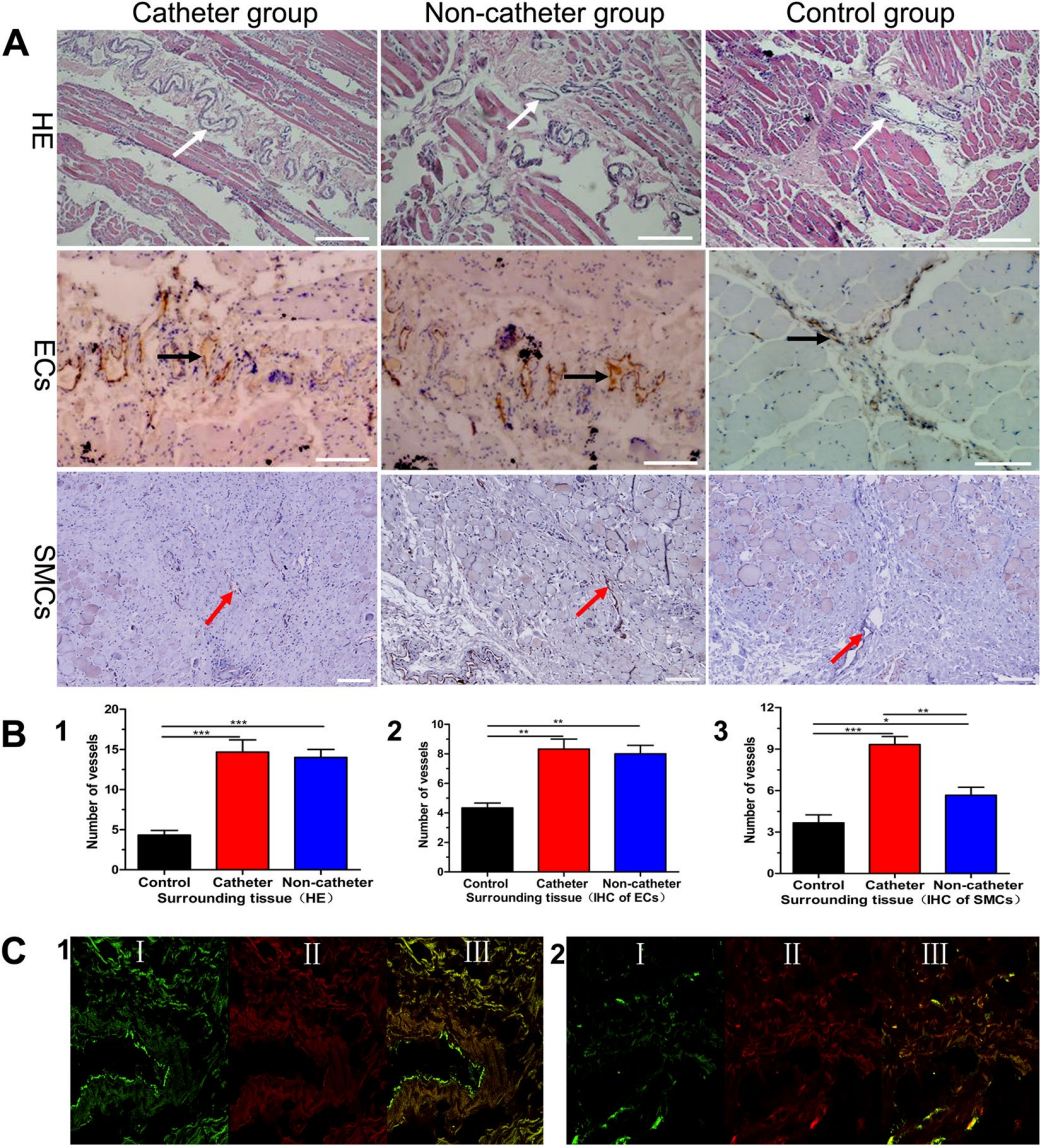
Angiogenesis of surrounding tissues in CG, NCG and NG. (**A**) Angiogenesis (HE, bar: 200 μm in CG/NCG, bar: 100 μm in NG), ECs (IHC, bar: 100 μm) and SMCs (IHC, bar: 100 μm). (**B**) Quantification, (1) New vessels formation (HE), (2) ECs positive vessels, (3) SMCs positive vessels. (**C**) Colocalization of MSCs and ECs (I green indicated MSCs, II red showed ECs, III merge), (1) Endothelium of TE artery and angiogenesis around TE artery in CG, (2) Endothelium of TE artery and angiogenesis around TE artery in NCG, blood vessels (White arrow), ECs positive vessel (Black arrow), SMCs positive vessel (Red arrow).

H&E and immunohistochemical staining showed that a large number blood vessels had formed in the tissues surrounding the TE arteries in both groups; however, there were few blood vessels in the tissues of the control group (Fig. 5A). The mean number of blood vessels was 15 ± 2 in the catheter group, 14 ± 1 in the non-catheter group, and 4 ± 1 in the control group (Fig. 5B_1_); the difference between the catheter and non-catheter groups was not statistically significant (P > 0.05). Immunohistochemical analysis for vWF found 8 ± 1 blood vessels in the catheter group vs. 8 ± 1 blood vessels in the non-catheter group and 4 ± 1 blood vessels in the control group (Fig. 5B_2_). Immunofluorescent analysis revealed more capillaries in the tissues around TE arteries in the catheter and non-catheter groups than in the control group. However, immunohistochemical staining for α-SMA indicated a significant difference between the two groups (9 ± 1 blood vessels in the catheter group vs. 6 ± 1 in the non-catheter group; P < 0.01); there were 4 ± 1 vessels in the control group (Fig. 5B_3_), which suggested that more mature blood vessels formed in the catheter group than in other groups. Some of the implanted MSCs in the collagen complex around the TE arteries were found to have differentiated into endothelial cells (Fig. 5C).

## Discussion

In this study, we employed the Vineberg procedure to implant a TE artery and, with sustained delivery of angiogenic growth factor and autologous MSCs, successfully established adequate blood supply to ischemic hind limbs of goats. Angiogenesis, vasculogenesis, and arteriogenesis were stimulated around the TE artery and a dense collateral circulation formed, with wide communication with the native vasculature.

When the distal part of the TE artery is ligated, preventing outflow, the blood within the artery will coagulate. To prevent this, we inserted a catheter into the artery and instituted continuous heparin infusion in one group. These TE arteries remained patent until the catheter was removed at the end of 1 month, whereas thrombosis and occlusion occurred within 7 days in the TE vascular grafts that did not receive the heparin infusion. Branches arising from the artery help maintain steady blood flow through the artery and thus prevent thrombosis. Following implantation of the TE artery, the sustained release of VEGF from the gelatin microspheres and the autologous MSCs within the CSs stimulated the growth of collateral vessels, which formed connections with the native microcirculation.

The importance of a good collateral circulation has long been known. The mechanisms governing the recruitment, growth, and proliferation of collateral vessels differs from those regulating angiogenesis and vasculogenesis^31^. In this study, for the development of a collateral circulation, new vessels had to sprout from the extra-anatomic TE artery wall, which was composed of natural ECM and therefore had ideal biocompatibility and mechanical retention characteristics. The decellularized vascular scaffold provided structural support and allowed for MSCs migration, growth, and differentiation and cellular ECM production^32^. It was expected that parts of scaffolds would degrade and allow collateral vessels development from the degraded locations^33^.

It is now accepted that neovascularization in the adult occurs by both angiogenesis and vasculogenesis^34^. In this study, angiogenesis and vasculogenesis were demonstrated around the TE artery and ischemic muscle. Our own preliminary studies and work by others have established that to achieve a mature vasculature, sustained release of cytokines and endothelial progenitor cells are needed at the ischemic site in a pattern that mimics the natural processes^35,36^. Previous studies have demonstrated that inflammatory cytokines stimulate the development of arteroles, venules, and capillaries. In this research, we used a collagen scaffold complex containing gelatin microspheres formulated for sustained VEGF release to promote angiogenesis. This system showed a stable VEGF release profile and performed better than collagen scaffold or gelatin microsphere alone.

In this study, autologous MSCs were used to achieve vasculogenesis. Vasculogenesis is the process of *in situ* formation of blood vessels from endothelial progenitor cells or angioblasts. It has been demonstrated that bone-marrow-derived endothelial precursor cells circulate in the peripheral blood and can incorporate into areas of neovascularization in the adult. Initially, mesenchymal cells differentiate *in situ* into primitive endothelial cells that generate a functioning vascular labyrinth^36,37^.

Our results show that the primitive vascular plexus subsequently develops into a complex, interconnecting network of mature blood vessels. Previous investigations have shown that MSCs can promote therapeutic angiogenesis and vasculogenesis, playing a key role in capillary endothelium formation and differentiating into smooth muscle cells or pericytes^38,39^. We hypothesized that autologous MSCs would not stimulate an immunologic reaction and that they would gather around the TE artery and facilitate the formation of arterial branches. We showed that the MSCs could differentiate into endothelial cells, which were involved in vasculogenesis around the TE arteries.

## Conclusion

In this study, we employed the Vineberg procedure in an ischemic hind limb model and successfully developed an extra-anatomic TE artery with a large number of collateral artery branches. We used MSCs and sustained-release GMs-VEGF formulations to stimulate angiogenesis, vasculogenesis, and arteriogenesis. Although some thrombosis occurred in the TE arteries 1 month after implantation, we have demonstrated the feasibility of the technique. Full development of the TE artery, with complete endothelialization and many outflow collaterals, may require many more weeks.
